# Tonsillar Lymphangiomatous Polyp Mimicking a Vallecular Cyst in an Adult: A Case Report

**DOI:** 10.1155/crot/6407488

**Published:** 2026-07-19

**Authors:** Mohammed A. Awadh, Manal Bin Manie, Sarah AlMansour, Latefa Alareek, Khaled Alhussinan, Faisal H. Zagzoog, Nourah Alkhudiri

**Affiliations:** ^1^ Department of Otolaryngology and Head and Neck Surgery, King Abdulaziz University Hospital, Jeddah, Saudi Arabia, kau.edu.sa; ^2^ College of Medicine, King Saud University, Riyadh, Saudi Arabia, ksu.edu.sa; ^3^ Department of Otolaryngology Head & Neck Surgery, Faculty of Medicine, King Abdulaziz University, Jeddah, Saudi Arabia, kau.edu.sa

**Keywords:** benign tumors, case report, lymphangioma, tonsillectomy, vallecular cyst

## Abstract

**Introduction:**

Tonsillar lymphangiomatous polyps are rare benign lesions characterized histologically by dilated lymphatic channels within a fibrous, adipose, or lymphoid stromal background. Although these lesions are more commonly reported in children and young adults, they are uncommon in older adults and may mimic other oropharyngeal or laryngeal lesions, including vallecular cysts.

**Case Presentation:**

We report the case of a 42‐year‐old previously healthy male who was incidentally found to have a mass at the base of the tongue. Preoperative clinical and radiologic assessment suggested a laryngeal vallecular cyst, as computed tomography demonstrated a well‐defined lesion occupying the left epiglottic vallecula with partial airway narrowing. However, intraoperative examination revealed that the lesion was pedunculated and originated from the left tonsil rather than the vallecula. The patient underwent surgical excision with left tonsillectomy. Histopathologic examination demonstrated a lesion covered by squamous epithelium and composed of multiple dilated lymphatic vascular channels within a fibrous stromal background. Immunohistochemical staining showed endothelial positivity for D2‐40, supporting lymphatic differentiation and confirming the diagnosis of a tonsillar lymphangiomatous polyp.

**Conclusion:**

This case highlights the diagnostic challenge of tonsillar lymphangiomatous polyps, particularly when a pedunculated tonsillar lesion projects toward the vallecula and clinically or radiologically mimics a vallecular cyst. Careful intraoperative assessment of the lesion’s attachment site is essential for accurate localization and diagnosis. Complete surgical excision remains both diagnostic and therapeutic.

## 1. Introduction

Benign tumors of the tonsil are generally uncommon and include squamous papillomas, angiomas, fibromas, myxomas, lipomas, and teratomatous cysts. Among these lesions, lymphatic tumors of the tonsil are considered particularly rare [1, 2]. Tonsillar lymphangiomatous polyps have been described using several terms in the literature, including polypoid lymphangioma of the tonsil, hamartomatous tonsillar polyp, lymphoid polyp, lymphangiectatic fibrous polyp, and tonsillar lymphangiomatous polyp, which may make their true incidence difficult to determine [3].

Histologically, these lesions are typically lined by squamous epithelium and contain a stromal component composed of loose or dense collagenous tissue, adipose tissue, lymphoid tissue, and dilated lymphatic channels [4]. They are most often reported in children, adolescents, and young adults, while presentation in older adults is uncommon [3, 5]. We present the case of a 42‐year‐old male with a mass initially suspected clinically and radiologically to represent a vallecular cyst but found intraoperatively to arise from the left tonsil and subsequently confirmed histopathologically as a tonsillar lymphangiomatous polyp.

## 2. Case Presentation

A 42‐year‐old previously healthy male presented to our clinic with a throat mass that had been present for approximately six months. The mass was first noticed incidentally during evaluation at a primary care center for fever and recurrent cough. On examination at that visit, a lesion was observed at the base of the tongue, and the patient was referred to our clinic for further assessment.

The patient was unable to determine whether the lesion had changed in size over time, as he had not directly visualized it himself. During the following 6 months, he experienced two mild choking episodes while eating and reported mild throat discomfort when lying down. He denied obstructive sleep apnea, dysphagia, odynophagia, voice changes, altered taste, recurrent tonsillitis, or recurrent throat infections. He reported increased salivation but no abnormal taste. He had no history of oral trauma, previous surgery, or prior intubation. He smoked cigarettes and used shisha approximately two to three times per week. There was no family history of autoimmune disease, head and neck malignancy, or laryngeal cancer.

On clinical examination, a smooth cystic‐appearing mass with no clearly visible base was seen protruding from the posterior tongue region. No palpable cervical lymphadenopathy was detected. Stroboscopic examination demonstrated a large, smooth‐surfaced lesion on the left side, compressing the epiglottis and extending superiorly. Both vocal cords were fully mobile.

A preoperative computed tomography scan of the neck demonstrated a small, well‐defined, fluid‐density lesion expanding the left epiglottic vallecula at the suprahyoid level. The lesion was adjacent to the proximal epiglottis, the base of the left hemitongue, the uvula, and the hypopharyngeal wall, with partial airway narrowing. It measured 3.4 × 2.0 × 1.6 cm and was interpreted radiologically as being suggestive of an epiglottic vallecular cyst with partial airway occlusion (Figure 1).

**FIGURE 1:**
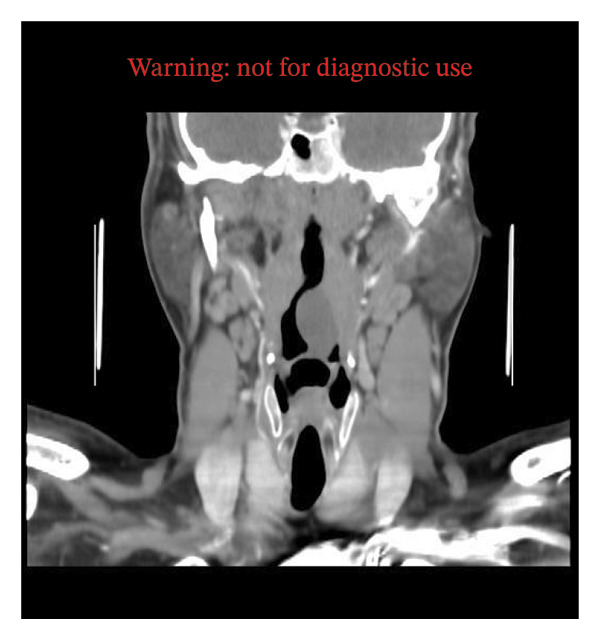
Preoperative computed tomography of the neck. Coronal CT images demonstrate a well‐defined, fluid‐density lesion occupying the left epiglottic vallecula and causing partial narrowing of the upper airway. The radiologic appearance was initially suggestive of a vallecular cyst.

The patient was scheduled for microlaryngoscopy, examination under general anesthesia, and excision of the mass. In the operating room, the patient was placed in the supine position, and general anesthesia was induced via an endotracheal tube. A shoulder roll was placed, and a Boyle–Davis mouth gag was positioned and suspended on a bipod stand. Intraoperative inspection demonstrated that the lesion was not arising from the vallecula; instead, it was pedunculated and originated from the left tonsil. A left tonsillectomy was performed using hot dissection with monopolar and bipolar diathermy, and the mass was removed en bloc with the left tonsil (Figure 2). Hemostasis was achieved, and there was no active bleeding at the end of the procedure. The patient tolerated the procedure well, was extubated safely, and was transferred to the recovery area in stable condition.

**FIGURE 2:**
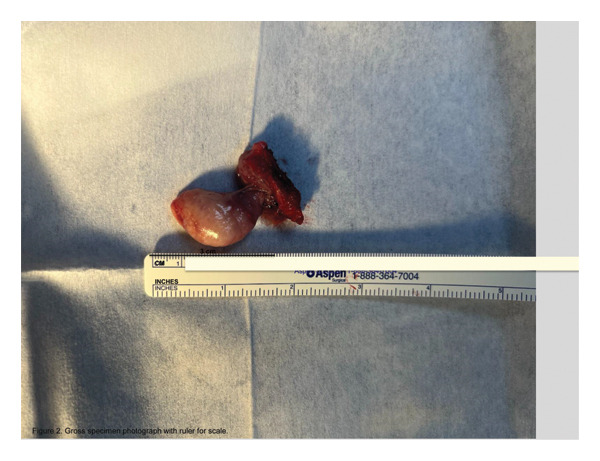
Intraoperative appearance of the lesion. Direct intraoperative visualization demonstrates a pedunculated mass arising from the left tonsil rather than the vallecula. The lesion was excised en bloc with the left tonsil.

Histopathologic examination showed a polypoid lesion covered by stratified squamous epithelium. The underlying stroma contained numerous dilated lymphatic vascular channels of variable size within a fibrous stromal background. Several of these channels contained lymphocytes and eosinophilic proteinaceous material, consistent with lymphatic spaces (Figure 3). Immunohistochemical staining demonstrated endothelial positivity for D2‐40, supporting lymphatic endothelial differentiation (Figure 4). These findings confirmed the diagnosis of a tonsillar lymphangiomatous polyp.

**FIGURE 3:**
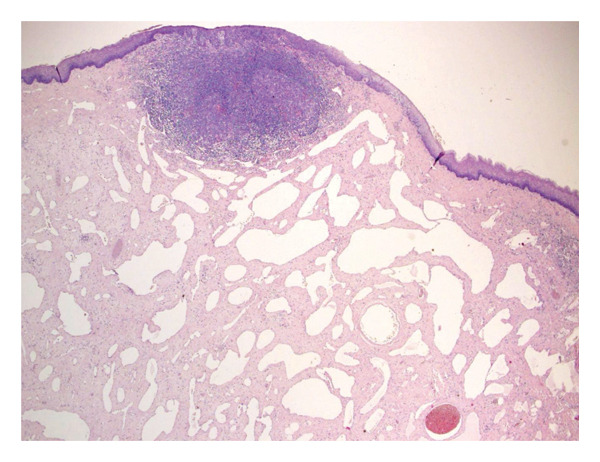
Histopathologic examination of the excised lesion. Hematoxylin and eosin–stained section shows a polypoid lesion covered by stratified squamous epithelium, with multiple dilated lymphatic vascular channels within a fibrous stromal background. Some channels contain lymphocytes and eosinophilic proteinaceous material.

**FIGURE 4:**
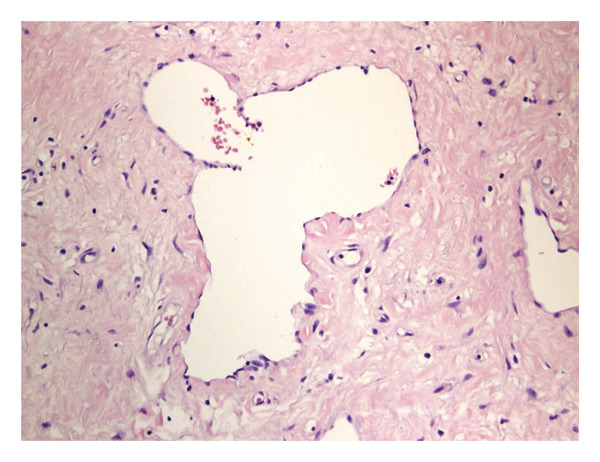
Immunohistochemistry for D2‐40. D2‐40 immunohistochemical staining highlights the endothelial lining of the dilated lymphatic channels, supporting lymphatic differentiation and confirming the diagnosis of a tonsillar lymphangiomatous polyp.

At the 2‐week postoperative follow‐up visit, the patient reported no active complaints. Flexible nasopharyngolaryngoscopy demonstrated a well‐healed surgical site, no evidence of residual or recurrent lesion, and bilaterally mobile vocal cords. The patient was subsequently discharged from routine follow‐up.

## 3. Discussion

A lymphangiomatous polyp of the tonsil is a benign hamartomatous lesion characterized by multiple dilated lymphatic channels within a stromal background that may contain fibrous tissue, lymphoid tissue, adipose tissue, or a combination of these elements. The lesion has been described using several names, including hamartomatous polyp of the tonsil, angiofibrolipoma, lymphangiomatous tonsillar polyp, and lymphangiectatic fibrous polyp [6]. This variability in terminology may contribute to underrecognition and difficulty in determining the true incidence of the condition.

The pathogenesis of tonsillar lymphangiomatous polyps remains uncertain, and several theories have been proposed. One theory suggests that chronic tonsillar inflammation may obstruct lymphatic drainage and lead to lymphatic dilatation. However, this explanation is limited by the fact that chronic tonsillitis is common, whereas lymphangiomatous polyps are rare [4, 7]. A second theory proposes that these lesions represent hamartomatous proliferations within tonsillar tissue rather than true neoplasms because their components are normally present in the tonsillar region but are arranged in an abnormal pattern [2]. A third theory suggests that failure of primordial lymphatic sacs to drain into the venous system may result in dilated lymphatic channels, which are characteristic of lymphangiomatous lesions [8].

The present case is clinically important because the lesion was initially interpreted as a vallecular cyst on both clinical and radiologic assessment, whereas intraoperative examination demonstrated its true origin from the left tonsil. This discrepancy was likely related to the pedunculated and mobile nature of the mass. Because the lesion projected medially and inferiorly toward the vallecular space, it appeared clinically to arise from the base of tongue or vallecula. On CT imaging, the mass occupied the left epiglottic vallecular region and partially narrowed the airway, which further supported the initial impression of a vallecular cyst. However, imaging may not always clearly demonstrate the site of attachment for a pedunculated or mobile oropharyngeal mass, particularly when the stalk is narrow or obscured by adjacent soft tissues.

In similar cases, several features may help distinguish a true vallecular lesion from a pedunculated tonsillar mass. A vallecular cyst typically arises from the mucosa of the vallecula or lingual surface of the epiglottis and is centered in relation to the vallecular space. In contrast, a pedunculated tonsillar lesion may project into the oropharynx or vallecula while maintaining a stalk or continuity with the palatine tonsil. Careful assessment of the lesion’s attachment site, mobility, relationship to the epiglottis, and continuity with tonsillar tissue is therefore essential. When preoperative localization remains uncertain, direct intraoperative visualization is particularly valuable, as it allows identification of the lesion’s stalk and definitive surgical planning.

Tonsillar lymphangiomatous polyps usually present during childhood, adolescence, or early adulthood. In a clinicopathologic series of 26 cases, the mean patient age was 25.2 years, and there was no clear sex predilection [2]. Reported symptoms include globus sensation, recurrent choking episodes, dysphagia, sore throat, and the sensation of a foreign body in the throat [2]. The present case is unusual because the patient was 42 years old and the lesion was initially suspected to be a vallecular cyst due to its apparent location and airway effect.

Histopathologic examination remains essential for diagnosis. Typical findings include a squamous epithelial covering overlying a stromal core composed of variable proportions of fibrous, adipose, and lymphoid tissue. Dilated lymphatic channels are a defining feature and may contain lymphocytes and eosinophilic proteinaceous material. Immunohistochemistry can support the diagnosis and help distinguish lymphangiomatous lesions from vascular lesions such as hemangiomas or arteriovenous malformations. CD31 may highlight vascular endothelium, while D2‐40 is a useful marker of lymphatic endothelial differentiation [9]. In the present case, the presence of dilated lymphatic channels and D2‐40–positive endothelial cells supported the diagnosis of a tonsillar lymphangiomatous polyp.

Complete surgical excision is recommended because it provides both definitive diagnosis and treatment, while also excluding other potentially aggressive lesions. The preferred approach is complete removal of the polyp together with the involved tonsil. Previously reported cases have not demonstrated recurrence or malignant transformation after complete excision. Nevertheless, the true incidence of lymphangiomatous or hamartomatous tonsillar polyps may be higher than reported because of underreporting and inconsistent terminology. This case contributes to the limited literature on adult presentations of tonsillar lymphangiomatous polyps and emphasizes the importance of considering a pedunculated tonsillar lesion in the differential diagnosis of a presumed vallecular cyst. As this is a single case report, the findings cannot be generalized to all patients with tonsillar lymphangiomatous polyps.

## 4. Conclusion

Tonsillar lymphangiomatous polyps are rare benign lesions that may present with nonspecific symptoms or be discovered incidentally. This case demonstrates that a pedunculated tonsillar lymphangiomatous polyp may mimic a vallecular cyst when it projects toward the vallecula and partially narrows the airway. Preoperative imaging is useful, but the site of origin may remain unclear when the lesion is mobile or pedunculated. Careful intraoperative evaluation of the attachment site is essential for accurate diagnosis and appropriate management. Complete surgical excision with tonsillectomy is both diagnostic and therapeutic, with no reported recurrence or malignant transformation after adequate removal.

## Funding

This study was not supported by any sponsor or funder.

## Conflicts of Interest

The authors declare no conflicts of interest.

## Data Availability

All data generated or analyzed during this study are included in this article. Further inquiries may be directed to the corresponding author.
